# Functional characterization of AtAAS as a 5-hydroxytryptophan decarboxylase linking serotonin formation to melatonin metabolism in *Arabidopsis thaliana*

**DOI:** 10.3389/fpls.2026.1961230

**Published:** 2026-09-15

**Authors:** Xiaohao Guo, Siyu Lin, Chuan Dong, Xinyu Huang, Hailian Hu, Baohai Li, Yuanyuan Tan, Qingyao Shu

**Affiliations:** 1 National Key Laboratory of Rice Breeding and Biology, and Zhejiang Provincial Key Laboratory of Crop Germplasm Innovation and Exploitation, The Advanced Seed Institute, Zhejiang University, Hangzhou, China; 2 State Key Laboratory for Development and Utilization of Forest Food Resources, Zhejiang A&F University, Hangzhou, China; 3 School of Marine Biology, Xiamen Ocean Vocational College, Applied Technology Engineering Center of Fujian Provincial Higher Education for Marine Resource Protection and Ecological Governance, Xiamen Key Laboratory of Intelligent Fishery, Xiamen, China; 4 State Key Laboratory of Soil Pollution Control and Safety, Zhejiang University, Hangzhou, China; 5 Ministry of Education (MOE) Key Laboratory of Environment Remediation and Ecological Health, College of Environmental and Resource Sciences, Zhejiang University, Hangzhou, China; 6 Zhejiang University - Wuxi Xishan Joint Modern Agricultural Research Centre, Zhejiang University, Hangzhou, China

**Keywords:** 5-hydroxytryptophan, aromatic L-amino acid decarboxylase, AtAAS, melatonin, N-acetylserotonin, serotonin

## Abstract

**Introduction:**

Serotonin and melatonin are plant signaling molecules involved in growth, stress responses, and redox balance. While animals produce serotonin via 5‑hydroxytryptophan (5‑HTP), plants are thought to use the tryptamine pathway. Although 5‑HTP exists in plants, its conversion to serotonin lacks a genetically defined enzyme.

**Methods:**

We performed phylogenetic and structural analyses of Arabidopsis aromatic aldehyde synthase (AtAAS), expressed recombinant AtAAS, and examined its 5-HTP decarboxylase activity in vitro. Enzyme kinetic parameters were determined, and endogenous 5-HTP levels in Arabidopsis were quantified. A T-DNA insertion mutant of AtAAS was further used to assess serotonin accumulation following 5-HTP feeding and to determine endogenous levels of serotonin, N-acetylserotonin, and melatonin under normal growth conditions.

**Results:**

AtAAS showed phylogenetic and structural similarities to animal aromatic amino acid decarboxylases and efficiently converted 5-HTP to serotonin in vitro (optimal pH 7.5, 55°C; Km = 1.806 mM, Vmax = 3.089 nmol/min/mg). Endogenous 5-HTP was detectable in Arabidopsis. Compared with wild-type plants, the AtAAS T-DNA insertion mutant exhibited reduced serotonin accumulation following 5-HTP feeding and decreased endogenous levels of downstream N-acetylserotonin and melatonin under normal growth conditions.

**Discussion:**

his discovery unveils enzymatic plasticity in plant serotonin biosynthesis and provides evidence for a previously unrecognized 5-HTP-dependent route in Arabidopsis.

## Introduction

Serotonin, a well-known neurotransmitter in animals, is also a recognized phytohormone in plants. It is widely distributed in plants and plays important roles in the regulation of growth and development, redox homeostasis, and responses to diverse biotic and abiotic stresses (Sun et al., 2025; Zhang et al., 2026; Mishra and Sarkar, 2023). In both plants and animals, serotonin is derived from a common precursor, tryptophan. It is widely accepted that in plants, tryptophan is first converted to tryptamine by tryptophan decarboxylase (TDC), then is hydroxylated to serotonin by tryptamine 5-hydroxylase (T5H); and in animals by contrast, tryptophan is first hydroxylated to 5-HTP by tryptophan hydroxylase (TPH), and then is decarboxylated to serotonin by aromatic L-amino acid decarboxylase (AADC) (Maffei, 2020; Back, 2021; Kang et al., 2007). However, 5-HTP was indeed identified in various plant tissues, and exceptionally high levels of 5-HTP were reported in the seeds of *Griffonia simplicifolia* (Bell and Fellows, 1966; Fellows and Bell, 1970; Puccetti et al., 2025). 5-HTP has also been detected in rice, particularly following genetic disruption or suppression of the conventional tryptamine-dependent serotonin pathway (Park et al., 2012, 2013). In addition, recombinant rice TDCs were shown to be capable of directly decarboxylating 5-HTP to serotonin in a pyridoxal 5′-phosphate-dependent way (Park et al., 2008). Furthermore, serotonin accumulation following 5-HTP feeding has also been observed in plants and in heterologous systems expressing rice TDC (Park et al., 2008). These pieces of evidence hint at the possible existence of an animal-like serotonin biosynthesis pathway in plants; however, a bona fide plant TPH responsible for the direct conversion of tryptophan to 5-HTP has not been conclusively identified.

As a model plant, Arabidopsis lacks functionally validated canonical TDC and T5H, and the enzymatic origin of serotonin in this species remains incompletely resolved (Hardeland, 2016; Negri et al., 2021). Hence, we questioned whether an animal-like serotonin biosynthesis pathway exists in Arabidopsis. To answer this question, we first searched AADC homologs and their encoding genes in Arabidopsis. Sequence- and structure-based phylogenetic analyses revealed that two previously characterized proteins, i.e., AtTyrDC and AtAAS, are closely related to human aromatic L-amino acid decarboxylase (HsAADC) and OsTDC2. AtTyrDC was reported to encode tyrosine decarboxylase (Lehmann and Pollmann, 2009), while AtAAS encodes aromatic aldehyde synthase, which converts phenylalanine and L-DOPA into phenylacetaldehyde and 3,4-dihydroxyphenylacetaldehyde, respectively (Gutensohn et al., 2011).

Given the generally distinct substrate preferences of plant TDCs and TYDCs for indole- and phenol-containing aromatic amino acids, respectively, AtTyrDC was considered a less likely candidate for 5-HTP decarboxylation (Facchini et al., 2000; Torrens-Spence et al., 2020). Previous biochemical analyses reported no detectable activity of wild-type AtAAS or its F338Y variant toward tryptophan or 5-HTP under the assay conditions employed (Torrens-Spence et al., 2013). However, the corresponding chromatographic data, quantitative activity measurements, and product-specific analyses for 5-HTP were not presented, leaving unresolved whether AtAAS activity toward 5-HTP was truly absent or merely below the detection threshold under those conditions. We therefore hypothesized that AtAAS could convert 5-HTP to serotonin while remaining functionally distinct from conventional plant TDCs that also catalyze tryptophan-to-tryptamine conversion, and tested this hypothesis by first assessing its activity toward tryptophan and then systematically characterizing 5-HTP conversion through direct product identification, optimization of catalytic conditions, and kinetic analysis. We further detected endogenous 5-HTP in Arabidopsis and compared wild-type and *AtAAS* T-DNA insertion mutant plants, hereafter referred to as *aas*, under untreated and 5-HTP-supplemented conditions to evaluate the contribution of AtAAS to 5-HTP-dependent serotonin formation and downstream N-acetylserotonin (NAS) and melatonin metabolism. Together, these analyses provide biochemical and in planta evidence that AtAAS mediates an animal-like 5-HTP-to-serotonin pathway that is functionally distinguishable from the classical tryptamine-dependent serotonin biosynthesis pathway.

## Results

### AtAAS is a candidate protein for 5-HTP decarboxylation

To identify candidate enzymes involved in serotonin-related decarboxylation reactions, the Arabidopsis proteome was searched for PLP-dependent aromatic amino acid metabolism-related enzymes using a Hidden Markov Model (HMM) profile of the Pyridoxal_deC domain (PF00282). Eventually, ten proteins were identified as candidates (**Table 1**).

**Table 1:** Genes annotated to encode proteins containing the Pyridoxal_deC domain in Arabidopsis.

Gene ID	Description
AT1G27980	dihydrosphingosine phosphate lyase
AT5G17330	glutamate decarboxylase
AT1G65960	glutamate decarboxylase 2
AT2G02000	glutamate decarboxylase 3
AT2G02010	glutamate decarboxylase 4
AT3G17760	glutamate decarboxylase 5
AT4G28680	L-tyrosine decarboxylase
AT1G43710	PLP-dependent transferases superfamily protein
AT2G20340	PLP-dependent transferases superfamily protein
AT3G17720	PLP-dependent transferases superfamily protein

To pinpoint the most likely candidates, sequence-based phylogenetic analysis was performed with human AADC (HsAADC) and rice TDCs. The results showed that AtAAS (*AT2G20340*) and AtTyrDC (*AT4G28680*) form a closely related Arabidopsis subclade positioned near OsTDC2 and HsAADC (**Figure 1A**). Structure-based comparison yielded a close relationship of AtAAS and AtTyrDC with HsAADC (**Figure 1B**). These results suggest that both AtAAS and AtTyrDC may possess enzymatic activities comparable to those of AADC.

**Figure 1:**
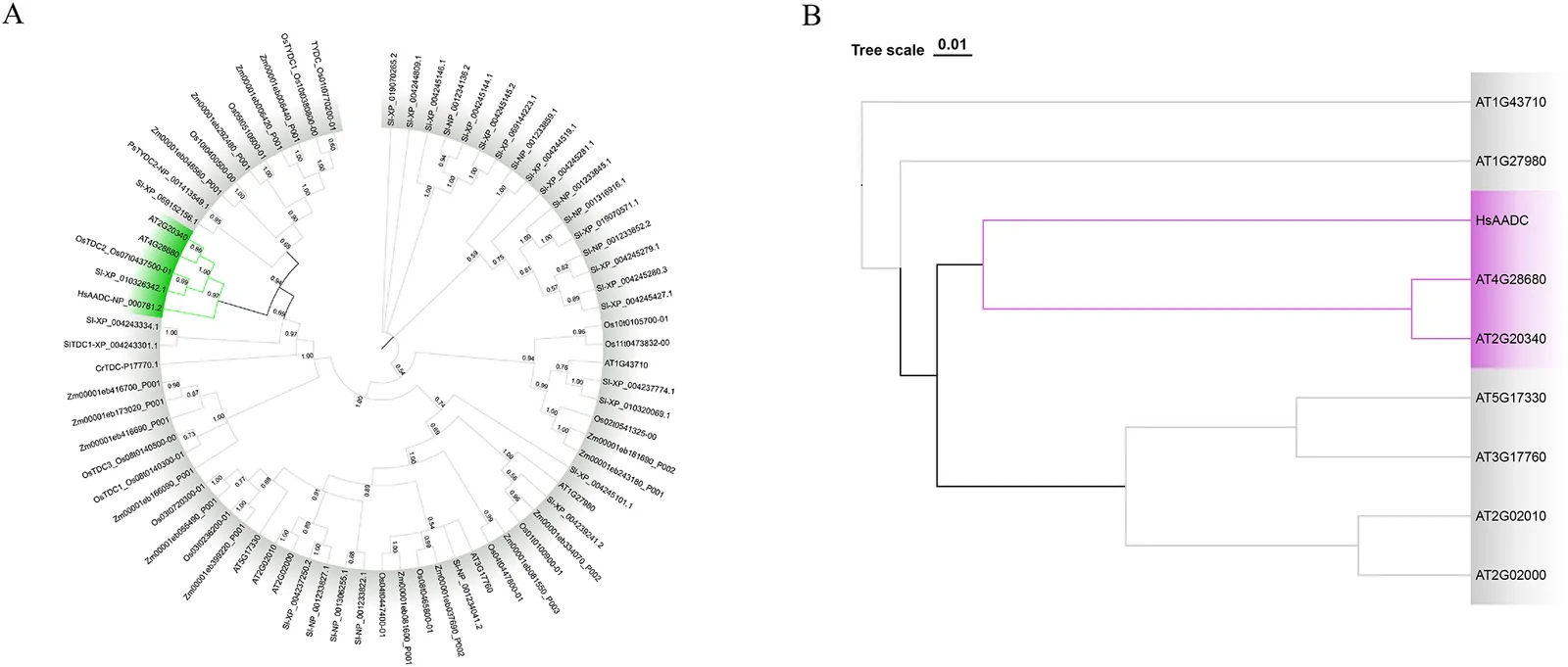
Phylogenetic relationships of Arabidopsis PLP-dependent aromatic amino acid metabolism-related proteins with other known AADC-family enzymes. **(A)** Maximum-likelihood phylogenetic tree of representative AADC enzymes from plants (including TDCs and TYDCs) and human AADC. **(B)** UPGMA clustering based on pairwise structural similarity among AADC-family proteins.

Previous biochemical and genetic studies have assigned the primary function of AtTyrDC to tyrosine decarboxylation and tyramine formation (Lehmann and Pollmann, 2009). Given that plant TDC- and TYDC-type enzymes generally exhibit pronounced substrate preferences, AtTyrDC was not considered the highest-priority candidate for the 5-HTP decarboxylation step. Although AtAAS had previously been characterized as an aromatic aldehyde synthase, its established reaction proceeds through an initial PLP-dependent amino acid decarboxylation step, followed by oxidative deamination and aldehyde formation (Gutensohn et al., 2011). AtAAS therefore retains the catalytic capacity required to initiate aromatic amino acid decarboxylation. Based on this mechanistic plausibility and functional distinction, AtAAS was selected for subsequent biochemical and in planta analyses.

### AtAAS directly converts 5-HTP to serotonin *in vitro*

To determine whether AtAAS can catalyze the conversion of tryptophan to tryptamine or 5-HTP to serotonin, recombinant AtAAS was produced (**Figure 2A**) and its enzymatic activities were assayed *in vitro* in the presence of PLP. Although AtAAS-mediated conversion of tryptophan to tryptamine was not detected in this study (**Supplementary Figure S1**), both HPLC and LC-MS/MS analyses of two independent samples showed that a significant amount of serotonin was produced from 5-HTP, demonstrating that AtAAS is capable of decarboxylating 5-HTP to serotonin (**Figures 2B, C**; **Supplementary Figure S2**).

**Figure 2:**
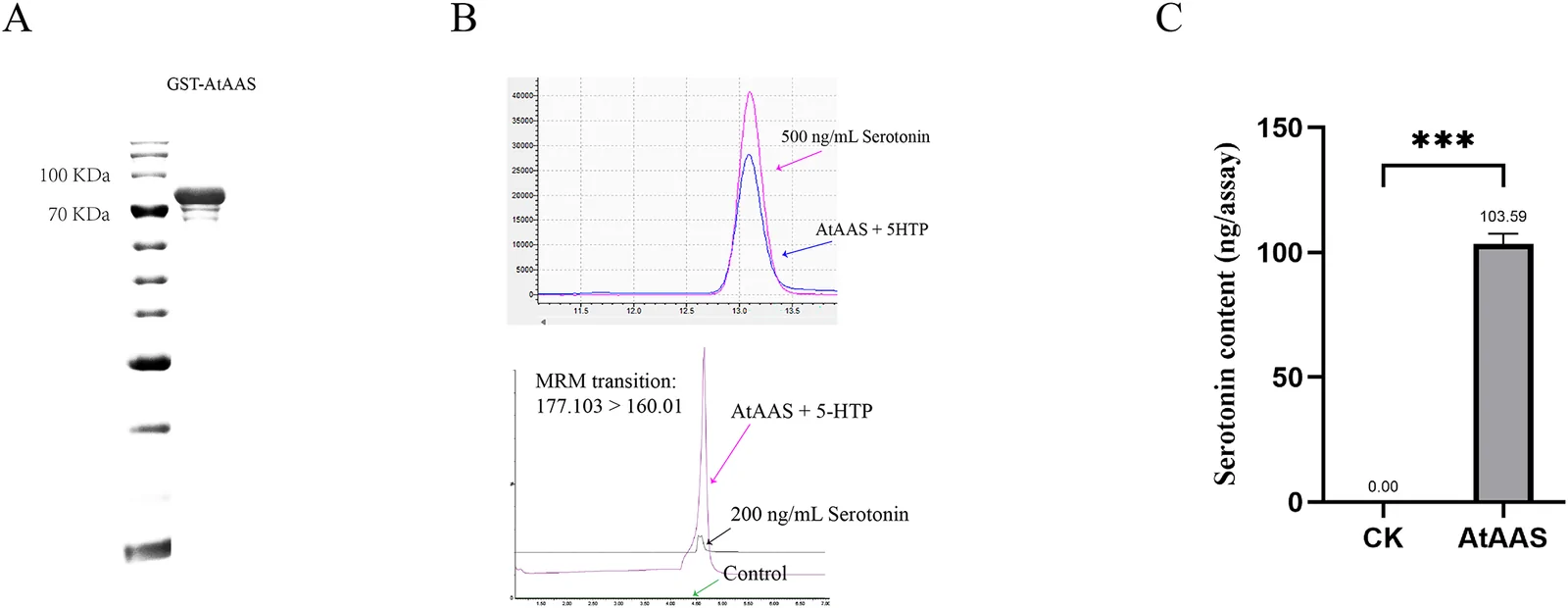
*In vitro* functional characterization of AtAAS. **(A)** SDS-PAGE of GST-tagged AtAAS recombinant protein. **(B)** Representative HPLC chromatograms showing serotonin production from 5-HTP by recombinant AtAAS. **(C)** Quantification of serotonin production by AtAAS under identical assay conditions. Serotonin produced from 5-HTP was measured after incubation of 50 μg recombinant AtAAS protein in 50 mM Tris-HCl buffer (pH 7.4) at 30 °C for 3 h. Data are shown as mean ± SD, n=3.

Because AtAAS belongs to the aromatic aldehyde synthase family (Torrens-Spence et al., 2013), the detection of AtAAS-catalyzed 5-HTP-to-serotonin conversion represents a previously unrecognized catalytic function.

### Enzymatic activity of AtAAS

To characterize the catalytic properties of AtAAS toward 5-HTP, the effects of temperature, pH, and the corresponding kinetic parameters were examined. Recombinant AtAAS displayed maximal activity at 55°C and pH 7.5 with 5-HTP as the substrate (**Figures 3A, B**). Under the optimized conditions, AtAAS exhibited an apparent *K*_m_ of 1.806 mM for 5-HTP, which was lower than the reported *K*_m_ for Phe (4.12 ± 0.39 mM) but higher than that for L-Dopa (0.55 ± 0.10 mM) (**Figure 3C**) (Gutensohn et al., 2011). Thus, AtAAS appears to bind 5-HTP with an intermediate apparent affinity relative to these two previously characterized substrates. Collectively, the present results demonstrate that AtAAS can efficiently catalyze reactions involving 5-HTP *in vitro*.

**Figure 3:**
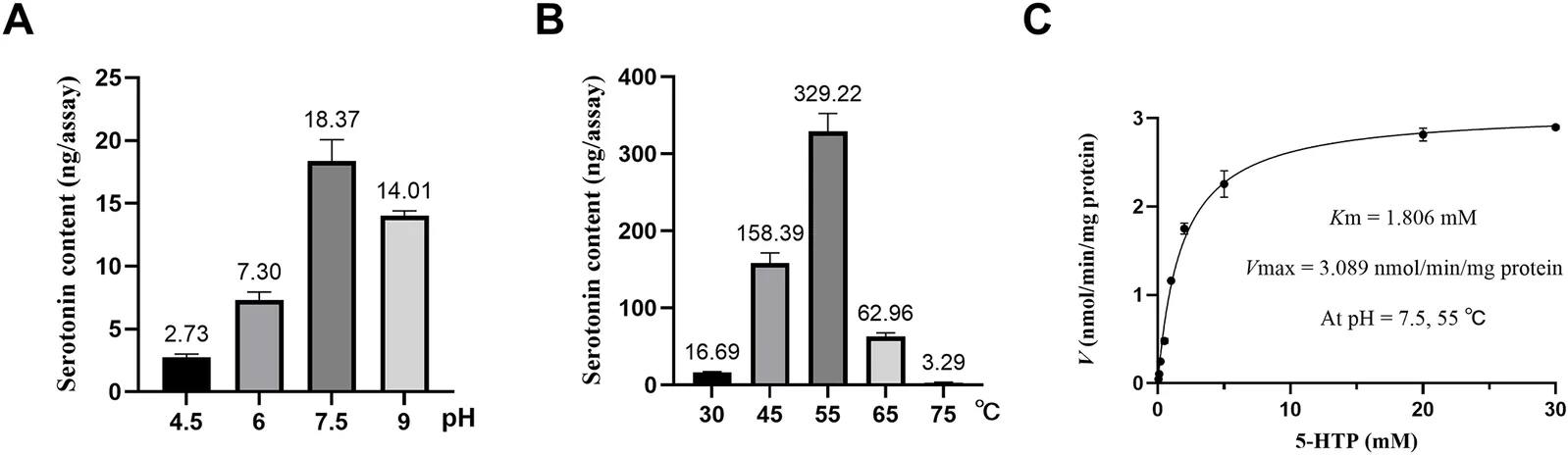
Enzymatic properties and kinetic characterizations of purified AtAAS. Effect of pH (4.5 - 9) **(A)** and temperature (30 °C - 75 °C) **(B)** on the enzymatic activity of AtAAS in catalyzing 5-HTP. Enzymatic activity was measured at various pH and temperatures conditions to determine the optimal reaction parameters in PBS buffer with 0.1 mM PLP for 1 hour. **(C)** Substrate affinity (*K*_m_) and maximum reaction velocity (*V*_max_) were determined for 5‐HTP using Michaelis-Menten kinetics. The reactions were performed in PBS buffer (pH = 7.5) with 0.1 mM PLP and a series of the concentrations of 5-HTP for 1 hour at 55 °C. Data are shown as mean ± SD, n=3.

### AtAAS is required for efficient 5-HTP-to-serotonin conversion in planta

In WT Arabidopsis plants, endogenous 5-HTP was readily detected, with a concentration of 8.1 ng/g in seedlings (**Supplementary Figure S3**). To determine whether AtAAS contributes to serotonin biosynthesis by decarboxylating 5-HTP *in vivo*, the serotonin level was first measured in Arabidopsis plants grown under the normal conditions. However, the serotonin levels were too low to be reliably quantified in both WT and *aas* mutant plants. To test whether AtAAS can utilize 5-HTP as a substrate in planta, the serotonin level was again assayed in WT and *aas* mutant seedlings, which were grown in medium supplemented with exogenous 200 μM 5-HTP. In these plants, serotonin was readily detected (**Figure 4A**), but its level was significantly lower in *aas* mutants than in wild-type plants (**Figure 4B**). These results strongly support the conclusion that AtAAS plays an important role in 5-HTP decarboxylation in Arabidopsis plants, thus suggesting the potential existence of an animal-like 5-HTP-dependent serotonin biosynthesis route.

**Figure 4:**
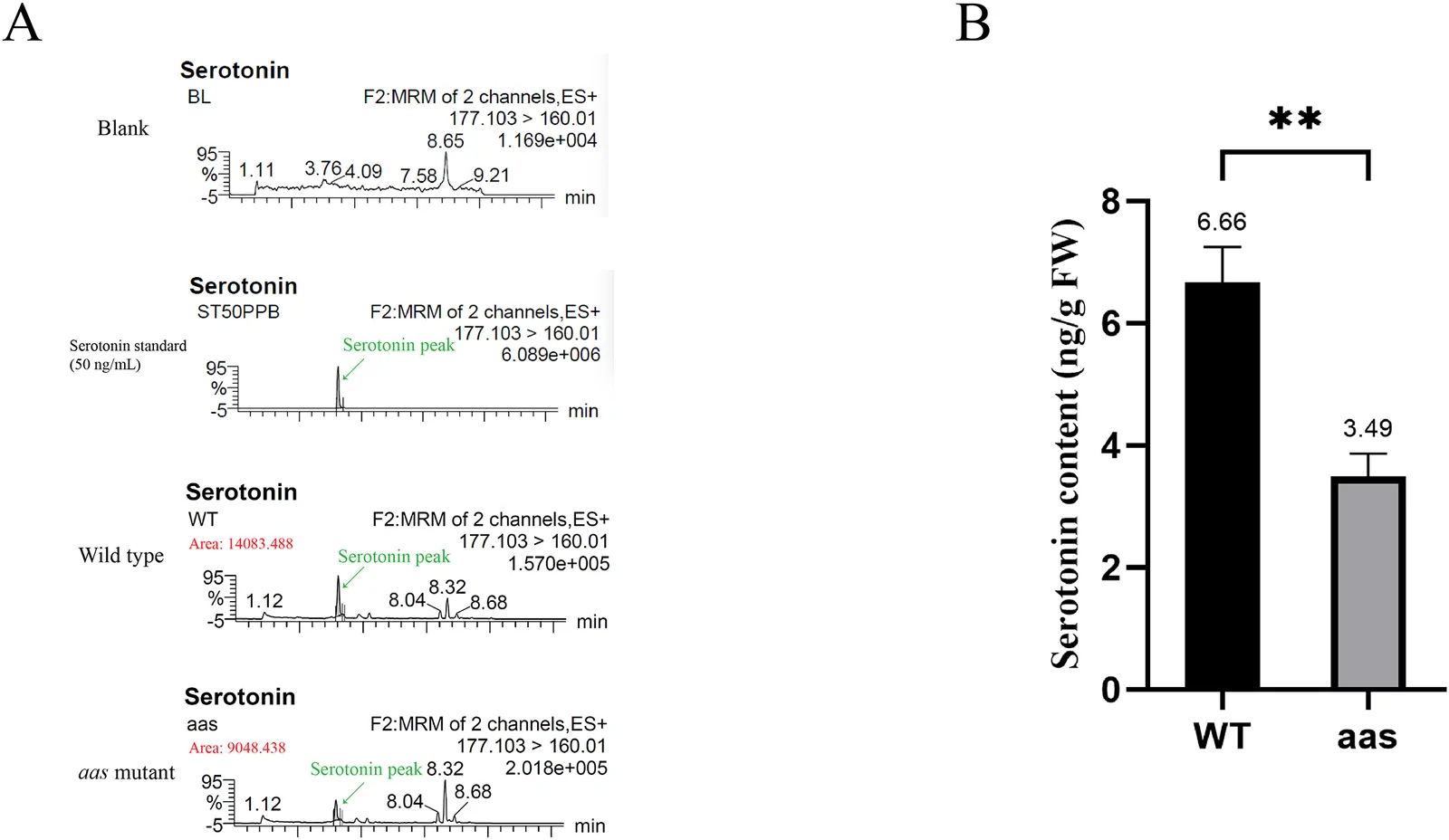
Serotonin accumulation in wild-type and *aas* mutant seedlings upon exogenous 5-HTP feeding. **(A)** Representative LC-MS/MS chromatograms of serotonin standard and its content in wild-type and *aas* mutant plants treated with 200 μM 5-HTP for 2 days. **(B)** Quantification of serotonin levels. Data are presented as mean ± SD (n = 4 independent biological replicates).

Notably, serotonin was still detectable in the *aas* mutant upon 5-HTP feeding, indicating that AtAAS is not the sole enzyme responsible for serotonin synthesis in planta, and that the plant tryptamine-dependent serotonin biosynthetic pathway may remain operational.

### The accumulation of downstream metabolites is reduced in the *aas* mutant

We next examined whether reduced serotonin availability in the *aas* mutant was accompanied by changes in metabolite levels in downstream melatonin pathway. Under normal growth conditions, NAS was consistently detected in wild-type plants, whereas its level was significantly reduced in the *aas* mutant (**Figure 5A**). This result links AtAAS-dependent serotonin supply to the metabolite pool available for downstream N-acetylation.

**Figure 5:**
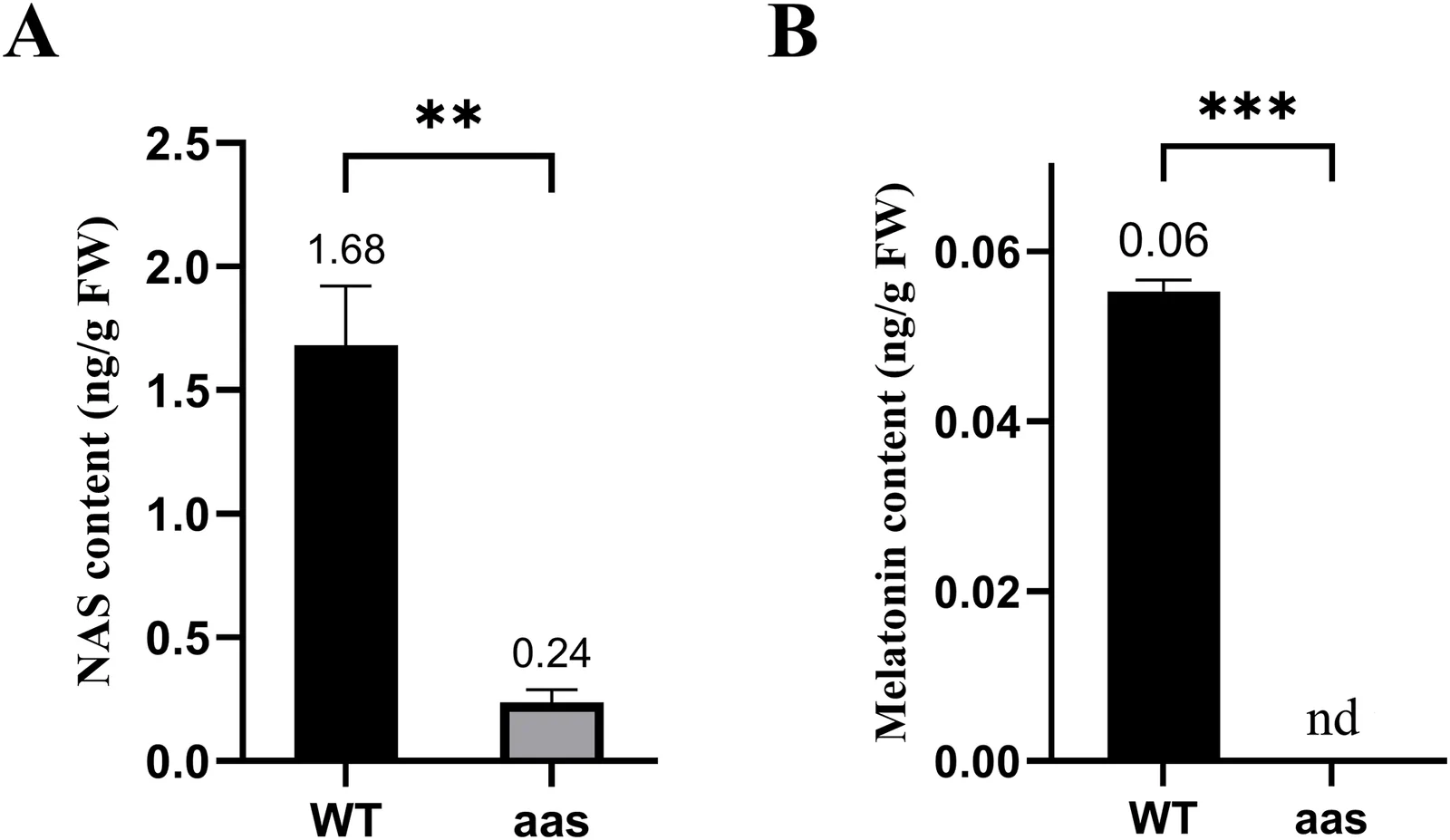
Metabolite accumulation in wild-type and *aas* mutant plants. **(A)** Quantification of N-acetylserotonin levels in wild-type (WT) and *aas* mutants under normal growth conditions. **(B)** Quantification of melatonin levels in wild-type (WT) and *aas* mutants under normal growth conditions. Data are presented as mean ± SD (n = 4 independent biological replicates).

Low but reproducible levels of melatonin were detected in wild-type plants. By contrast, melatonin levels in the *aas* mutant were reduced to undetectable levels (**Figure 5B**), indicating a severe impairment of melatonin-related accumulation. The concurrent decreases in 5-HTP-dependent serotonin production, basal NAS content, and melatonin levels collectively implicate AtAAS in the downstream serotonin–NAS–melatonin metabolic pathway.

### Arabidopsis accessions harbor common and rare AtAAS protein variants

To place the newly identified AtAAS activity in a natural-variation context, we analyzed variant-effect annotations for AT2G20340.1 using TAIR10 as the reference. The dataset contained 20,397 accession-variant records from 957 accessions with recorded variation in the AtAAS region, representing 258 unique genomic positions. These included 32 unique missense positions, 43 synonymous positions, 147 intronic positions, 31 3′-UTR positions, 15 splice-region positions, five 5′-UTR positions, two predicted splice-donor positions, and one predicted 5′-UTR premature-start-codon-gain position (**Figures 6A, B**).

**Figure 6:**
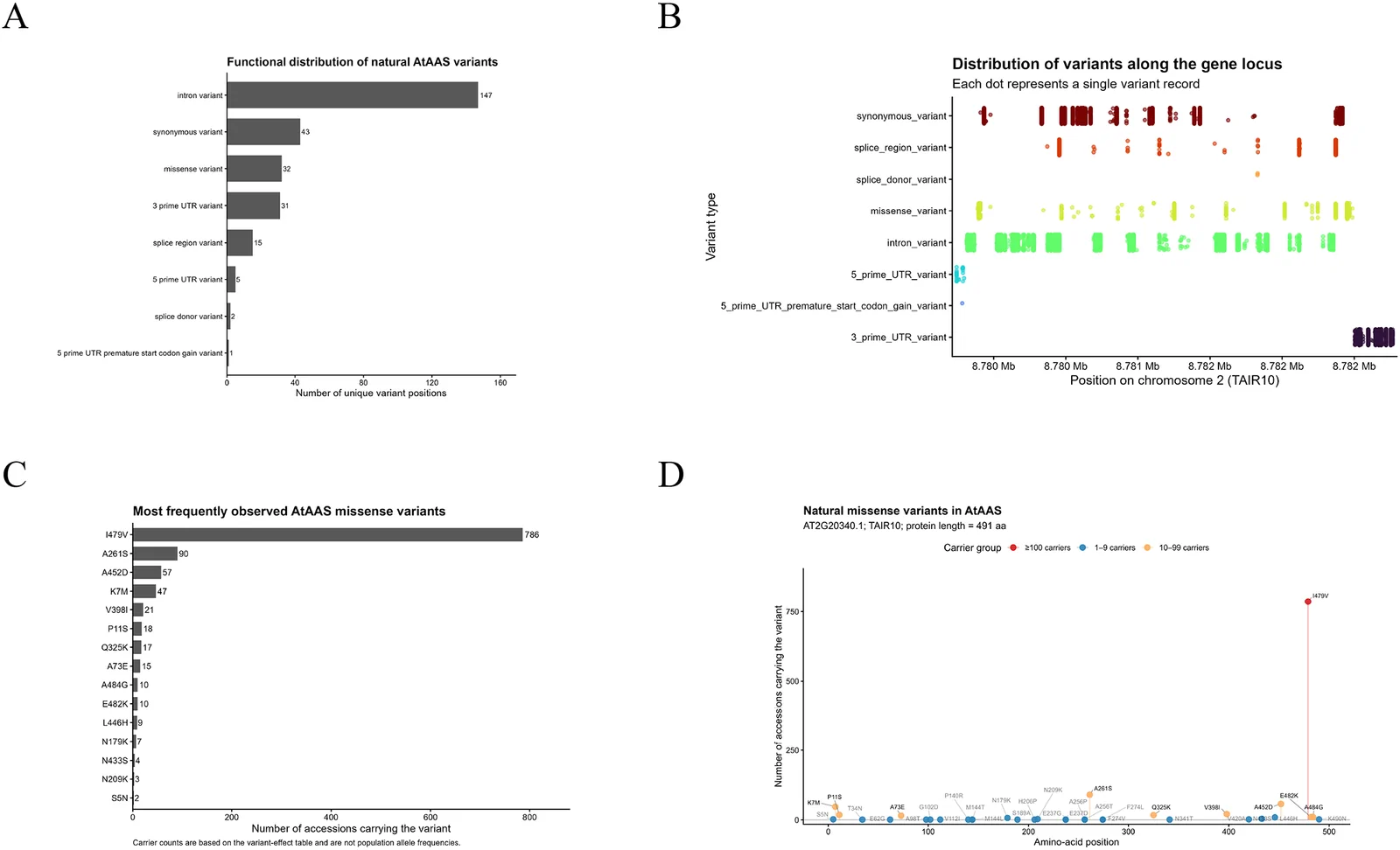
Natural variation in AtAAS across Arabidopsis accessions. **(A)** Variant type distribution. **(B)** Jitter plot of variant positions along the AtAAS genomic sequence. **(C)** Carrier counts for recurrent missense substitutions; I479V is the most frequent (786 accessions). **(D)** Jitter plot of missense variants mapped onto the 491-amino-acid AtAAS protein, showing uneven distribution with I479V as the predominant variant.

Mapping missense substitutions onto the 491-amino-acid protein revealed an uneven distribution of carrier counts (**Figures 6C, D**). I479V was the most frequently recorded substitution and was present in 786 accessions with variant records. Other recurrent substitutions included A261S (90 accessions), A452D (57), K7M (47), V398I (21), P11S (18), Q325K (17), A73E (15), and E482K and A484G (10 each). Most remaining substitutions were observed in only a small number of accessions.

This analysis identifies naturally occurring AtAAS variants and their population frequencies, providing a resource for future studies. However, biochemical validation is required to determine whether any of these substitutions affect enzymatic activity or downstream metabolism.

## Discussion

### AtAAS possesses a previously unrecognized 5-HTP decarboxylation activity

The central finding of this study is that AtAAS directly catalyzes the conversion of 5-HTP to serotonin. AtAAS was previously characterized as an aromatic aldehyde synthase that converts selected aromatic amino acids into their corresponding aldehydes through coupled decarboxylation and oxidative deamination. Our findings expand its known catalytic repertoire by showing that AtAAS can generate the corresponding amine product, serotonin, from 5-HTP under the conditions tested.

This observation is consistent with a model of substrate-dependent catalytic partitioning. When 5-HTP serves as the substrate, AtAAS may complete PLP-dependent decarboxylation but fail to proceed efficiently through the subsequent oxidative deamination and aldehyde-forming steps, resulting in the release of serotonin. This suggests that the catalytic outcome of AtAAS is not strictly constrained by its conventional annotation as an aromatic aldehyde synthase, but may instead depend on the chemical properties of the substrate. More broadly, this finding highlights the functional plasticity of plant AADC-related enzymes that share a common PLP-dependent catalytic framework. Given that AtAAS exhibits substantially higher catalytic efficiency toward phenylalanine (and L-DOPA) than toward 5-HTP, and endogenous phenylalanine levels far exceed those of 5-HTP in Arabidopsis, AtAAS likely functions primarily as an aromatic aldehyde synthase utilizing phenylalanine *in vivo* (Gutensohn et al., 2011). The 5-HTP decarboxylation activity should therefore be viewed as a contributing pathway rather than the major route. This is particularly relevant because Arabidopsis lacks a functionally validated TDC and has extremely low tryptamine levels, so even moderate 5-HTP decarboxylation could contribute meaningfully to serotonin formation, consistent with the reduced serotonin, NAS, and melatonin levels in the *aas* mutant.

Although recombinant AtAAS exhibited optimal activity at 55°C *in vitro*, well above the normal growth temperature of Arabidopsis (22–23°C), we interpret this high apparent optimum primarily as a property of the heterologously expressed GST-tagged protein and the specific assay conditions, rather than as a reflection of its physiological operating temperature. Notably, a similar phenomenon has been observed for the downstream enzyme AtSNAT2, which also displays optimal temperatures of 45°C and 55°C *in vitro* (Lee et al., 2019). From a physiological perspective, melatonin is a well-known stress metabolite that accumulates under heat stress and contributes to thermotolerance in plants (Khan et al., 2026; Sopory, 2026). SNAT itself has been regarded as a heat-resistant enzyme, and its unusually high temperature optimum *in vitro* may reflect the need to maintain melatonin synthesis under high-temperature conditions (Guo et al., 2024; Lee and Back, 2026). By analogy, the high apparent optimum of AtAAS might similarly ensure that serotonin supply - and consequently melatonin production - remains functional during heat stress episodes to support plant defense responses. Whether this property has direct adaptive significance in planta warrants further investigation. Nonetheless, the standard activity assays were performed at 30°C, where AtAAS retains substantial activity, confirming its catalytic competence at physiologically relevant temperatures.

### Genetic and metabolic evidence supports an AtAAS-mediated animal-like 5-HTP-to-serotonin step in Arabidopsis

Our study provides a clear genetic model to dissect the 5-HTP-to-serotonin pathway independently from the classical tryptamine pathway, a distinction that was previously difficult to establish in plants like rice (Back, 2021). The direct 5-HTP decarboxylation activity of AtAAS establishes the biochemical basis of the reaction, but its physiological relevance in planta requires additional evidence. The detection of endogenous 5-HTP in Arabidopsis provides a naturally occurring substrate pool for the 5-HTP-to-serotonin reaction. More importantly, following exogenous 5-HTP supplementation, serotonin accumulation was significantly lower in the *aas* mutant than in wild-type plants. An alternative interpretation is that exogenous 5-HTP could be converted back to tryptophan and subsequently enter the classical tryptamine-dependent pathway to produce serotonin. However, if this were the primary route, wild-type and *aas* mutant plants would be expected to show similar serotonin accumulation after 5-HTP feeding, because both genotypes would rely on the same downstream TDC-dependent pathway. Our observation that serotonin levels were significantly lower in the *aas* mutant (**Figure 4B**) therefore does not support this alternative explanation. Moreover, recombinant AtAAS did not catalyze tryptophan-to-tryptamine conversion *in vitro* (**Supplementary Figure S1**), indicating that disruption of AtAAS would not directly impair the classical route. Together, these results link the *in vitro* activity of recombinant AtAAS to an in planta metabolic phenotype and supports a role for AtAAS in 5-HTP-dependent serotonin formation in Arabidopsis.

This finding supports the existence of an animal-like 5-HTP-to-serotonin step in planta. Rice TDC has been shown to catalyze both the decarboxylation of tryptophan to tryptamine and that of 5-HTP to serotonin. Because the same enzyme mediates both reactions, direct 5-HTP decarboxylation cannot be experimentally and genetically distinguished from the classical tryptamine-dependent pathway. By contrast, neither previous studies nor the present work detected AtAAS-mediated conversion of tryptophan to tryptamine under the conditions tested. Therefore, the reduced serotonin accumulation observed in the *aas* mutant after 5-HTP supplementation is unlikely to result from direct disruption of the classical tryptophan-to-tryptamine reaction and instead supports an AtAAS-mediated 5-HTP-to-serotonin step that is functionally distinguishable from the classical plant pathway.

Thus, this study not only identifies a previously unrecognized 5-HTP decarboxylation activity but also provides support for the physiological relevance of this animal-like reaction step in Arabidopsis. Importantly, the present findings establish the second step of an animal-like route, namely 5-HTP-to-serotonin conversion, rather than the complete tryptophan-to-5-HTP-to-serotonin pathway.

The persistence of detectable serotonin in the *aas* mutant is also informative. It indicates that AtAAS is not the sole source of serotonin in Arabidopsis and that the classical tryptamine-dependent route, other low-activity AADC-related enzymes, or as-yet unidentified parallel pathways may continue to contribute to serotonin formation. The most appropriate interpretation is therefore that AtAAS makes an important contribution to 5-HTP-dependent serotonin formation rather than determining total serotonin biosynthesis.

We recognize an important limitation regarding the interpretation of the *aas* mutant phenotypes. The observed alterations in serotonin and downstream metabolites could, in principle, reflect indirect effects of AtAAS disruption on broader aromatic amino acid metabolism, rather than a direct reduction in 5-HTP decarboxylation activity in planta. We have not compared the aas mutant with mutants defective in the classical tryptamine-dependent pathway, nor have we constructed a double mutant to test whether both routes account for all serotonin production in Arabidopsis. Such genetic experiments are currently beyond the scope of this study, in part because the canonical TDC gene(s) responsible for the classical pathway in Arabidopsis have not yet been conclusively identified. We therefore interpret our findings as supporting an AtAAS-mediated contribution to 5-HTP-dependent serotonin formation, rather than establishing a complete and genetically independent pathway.

### The AtAAS-dependent 5-HTP-to-serotonin reaction affects downstream NAS and melatonin-related metabolism

Under normal growth conditions, NAS levels were significantly reduced in the *aas* mutant, indicating that the role of AtAAS is not restricted to serotonin biosynthesis via 5-HTP decarboxylation. NAS is the direct N-acetylated product of serotonin, and its reduction indicates that changes in AtAAS-dependent serotonin supply are transmitted to the downstream metabolic pool (Byeon et al., 2014; Lee et al., 2014, 2019). Furthermore, melatonin level was also markedly reduced in the *aas* mutant (**Figure 5**), which indicates that the AtAAS-mediated 5-HTP-to-serotonin step affects not only serotonin itself but also the availability of substrate for subsequent melatonin biosynthesis and metabolism. Collectively, these results demonstrate that AtAAS-mediated 5-HTP decarboxylation affects serotonin availability and consequently influences downstream NAS and melatonin-related metabolism.

### AtAAS reveals pathway parallelism and enzymatic plasticity in Arabidopsis serotonin biosynthesis

By linking a previously characterized aromatic aldehyde synthase to an animal-like 5-HTP-to-serotonin step in Arabidopsis, this study expands the established biological function of AtAAS. The findings indicate that plant AADC-related enzymes can share a common PLP-dependent catalytic chemistry while producing different reaction outcomes depending on substrate structure, active-site configuration, and catalytic environment. The ability of AtAAS to utilize 5-HTP illustrates the potential catalytic plasticity of this enzyme family.

At the pathway level, our results support the existence of an animal-like 5-HTP-to-serotonin step operating in parallel with the classical tryptamine-dependent pathway in Arabidopsis. Unlike rice TDC, which catalyzes both tryptophan-to-tryptamine and 5-HTP-to-serotonin reactions, AtAAS provides a functionally distinguishable entry point for dissecting direct 5-HTP decarboxylation. This suggests that plant serotonin biosynthesis may not be restricted to a single fixed route, but may instead rely on distinct enzymatic strategies depending on species, tissue, developmental stage, and metabolic context.

Given that tryptophan and tryptamine serve as common precursors for both serotonin and IAA biosynthesis, the diversion of metabolic flux through an AtAAS-mediated 5-HTP decarboxylation branch could, in principle, influence IAA homeostasis in Arabidopsis. Moreover, serotonin itself shares structural similarity with IAA and has been shown to act as a natural auxin inhibitor, affecting root system architecture (Pelagio-Flores et al., 2011; Amiri, 2026). Thus, altered serotonin levels resulting from AtAAS disruption might not only reflect changes in indolamine metabolism but could also indirectly modulate auxin-related processes. However, our present study did not measure IAA levels or directly assess metabolic flux distribution between these pathways, and this possibility remains speculative at this stage.

Several limitations remain. First, the upstream enzyme responsible for hydroxylating tryptophan to 5-HTP in Arabidopsis has not yet been identified, and the complete animal-like tryptophan-to-5-HTP-to-serotonin pathway therefore remains unresolved. Second, the TDC gene responsible for the classical tryptamine-dependent pathway in Arabidopsis has not yet been identified. Therefore, the extent to which residual serotonin in the *aas* mutant originates from this classical route remains unknown. Finally, The natural variation data presented in this study are exploratory and do not establish functional differences among AtAAS alleles. Future studies are needed to assess whether the identified variants, particularly I479V, differentially affect 5-HTP decarboxylase activity or serotonin/melatonin levels.

## Conclusion

This study reveals a previously unrecognized 5-HTP decarboxylation activity of AtAAS and provides biochemical, genetic, and metabolic evidence for an animal-like 5-HTP-to-serotonin step in Arabidopsis. Recombinant AtAAS converted 5-HTP to serotonin *in vitro*, whereas the aas mutant accumulated significantly less serotonin than wild-type plants following 5-HTP supplementation. Under untreated growth conditions, the mutant also exhibited reduced NAS and melatonin levels. These findings support AtAAS-mediated serotonin formation that is functionally distinguishable from the classical tryptamine-dependent pathway. Residual serotonin in the *aas* mutant suggests additional parallel or compensatory routes exist. Overall, our findings provide direct in planta evidence for an animal-like 5-HTP-to-serotonin step in Arabidopsis and reveal greater diversity in plant serotonin biosynthesis than the conventional single-pathway model.

## Proposed model

Based on the findings of this study, we propose a working model for serotonin biosynthesis in Arabidopsis comprising two functionally distinguishable routes (**Figure 7**). In the classical plant tryptamine-dependent pathway, tryptophan is first decarboxylated to tryptamine, which is subsequently hydroxylated to serotonin. This second step is shown as a dashed green arrow because the corresponding tryptamine 5-hydroxylase gene has not yet been identified in Arabidopsis. In parallel, an animal-like 5-HTP-dependent route is proposed. In this route, tryptophan is first hydroxylated to 5-HTP. This upstream step is shown as a dashed blue arrow because the responsible TPH-like enzyme has not yet been identified in plants. The present study supports a role for AtAAS in mediating the subsequent conversion of 5-HTP to serotonin. Recombinant AtAAS directly converted 5-HTP to serotonin *in vitro*, endogenous 5-HTP was detected in Arabidopsis, and the *aas* mutant showed significantly reduced serotonin accumulation following 5-HTP supplementation. In addition, NAS and melatonin-related accumulation were decreased in the mutant under normal growth conditions.

**Figure 7:**
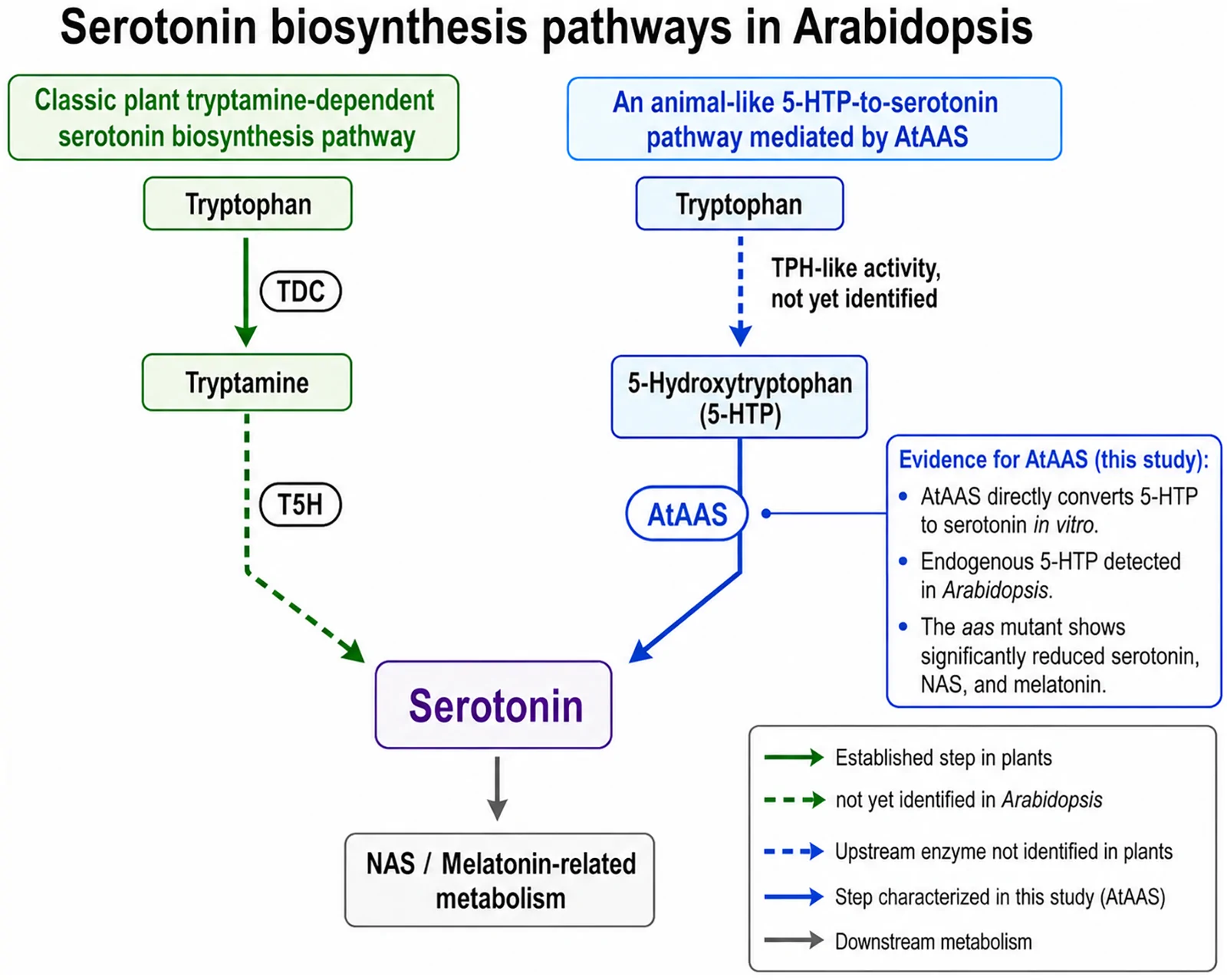
Working model of serotonin biosynthesis and downstream metabolism in Arabidopsis.

The convergence of these two routes at serotonin suggests that Arabidopsis may employ both the classical plant tryptamine-dependent pathway and a functionally distinguishable animal-like 5-HTP-to-serotonin step. This model therefore supports the existence of an AtAAS-mediated animal-like 5-HTP-to-serotonin step in Arabidopsis, while the enzymes responsible for upstream tryptophan hydroxylation and classical tryptamine hydroxylation remain unresolved. More broadly, animal and plant serotonin biosynthesis have traditionally been regarded as proceeding through distinct routes. The ability of the plant enzyme AtAAS to catalyze the 5-HTP-to-serotonin reaction demonstrates that a step previously associated primarily with the animal pathway can also operate in plants. This finding challenges the conventional separation between animal-type and plant-type serotonin biosynthetic routes and reveals greater flexibility in plant serotonin biosynthesis than previously recognized.

## Materials and methods

### Plant materials and growth conditions

*Arabidopsis thaliana* ecotype Columbia-0 (Col-0) and the *aas* mutant (T-DNA insertion mutant line N672304) were used in this study. The *aas* mutant line was obtained from AraShare (http://www.arashare.cn). The *aas* mutant (N672304) has been characterized as a knockdown allele, carrying a T-DNA insertion in exon 13 of AtAAS (accompanied by a 31-bp deletion) that results in a transcript lacking its authentic 3′ end and an approximately 55% reduction in mRNA levels (Gutensohn et al., 2011). The *aas* mutant line was genotyped by PCR using gene-specific primers flanking the insertion site and a T-DNA border primer. Homozygous mutant plants were identified and used for metabolite analysis. Seeds were surface-sterilized and sown on ½-strength Murashige and Skoog (MS) medium (Murashige and Skoog, 1962) solidified with 0.8% agar. After stratification at 4°C for 2 days, the plates were transferred to a growth chamber maintained at 22°C, 60% relative humidity, and a 16 h light/8 h dark photoperiod. Seedlings were grown on the ½-MS plates for 10 days. They were then transferred to ½-strength Yamagami nutrient solution (Shikanai et al., 2015) adjusted to pH 5.5 for a 4-day adaptation period. Subsequently, the seedlings were moved to full-strength Yamagami nutrient solution (pH 5.5) and grown for an additional 6 days. After this period, the nutrient solution was replaced with full-strength Yamagami nutrient solution containing 200 μM 5-hydroxytryptophan (5-HTP), and the plants were treated for 2 days. The above-ground tissues were then harvested, immediately frozen in liquid nitrogen, and stored at –80°C until further analysis.

### Bacterial strains, vectors, and chemicals

*Escherichia coli* strain DH5α was purchased from Tsingke Biotechnology (Beijing, China) and used for plasmid propagation. The expression strain *E. coli* OverExpress C43(DE3) was obtained from Weidi Biotechnology (Shanghai, China). The pET-41b vector was used for recombinant protein expression.

Chemicals and standards: tryptophan, tryptamine, tryptone, yeast extract, sodium chloride, PBS buffer, and Isopropyl-beta-D-thiogalactopyranoside (IPTG) were purchased from Sangon Biotech (Shanghai, China). Pyridoxal phosphate (PLP) was obtained from Shanghai Yuanye Bio-Technology (Shanghai, China). 5-Hydroxytryptophan (5-HTP), serotonin, N-Acetyl-5-Hydroxytryptamine and melatonin were purchased from Aladdin Biochemical Technology (Shanghai, China).

### Identification of AADC-family candidate genes in Arabidopsis and related species

Protein sequence database and annotation files were downloaded from the Ensembl Plants database for *A. thaliana*. The hidden Markov model (HMM) profile Pyridoxal_deC.hmm (PF00282) was used to search the protein sequences, and candidate genes were screened with an e-value threshold of < 0.01. The amino acid sequences of the candidate genes were aligned using CLUSTALW 2.0, and the resulting alignment was used to construct a specific Pyridoxal_deC.hmm HMM profile. This new profile was then used to re-search the protein sequences, again with an e-value threshold of < 0.01. The newly identified candidate genes were considered to be the AADC family members. The presence of the Pyridoxal-dependent decarboxylase conserved domain in the candidate genes was confirmed using NCBI’s CDD and the InterPro database.

### Phylogenetic tree construction and structure-based hierarchical clustering

Amino acid sequences were aligned using MUSCLE in MEGA12. A maximum-likelihood (ML) phylogenetic tree was constructed with the appropriate substitution model. We referred to the method of Huang et al. (2023) (Discovery of deaminase functions by structure-based protein clustering) to construct a structure-based hierarchical clustering tree. Protein structures were predicted using ESMFold (Evolutionary-scale prediction of atomic-level protein structure with a language model) and saved in PDB format. Pairwise structural alignments were performed using TMalign (TM-align: a protein structure alignment algorithm based on the TM-score). For each protein pair, TM-scores were extracted from the TMalign output, and the maximum TM-score was used as the pairwise structural similarity score. The similarity score was converted into a structural distance using D = 1−S, where S represents the TM-score. The resulting symmetric distance matrix was used to construct a structure-based hierarchical clustering tree using the UPGMA algorithm implemented in the Bio.Phylo.TreeConstruction module of Biopython. The final tree was exported in Newick format for visualization. The tree was visualized and beautified using tvBOT (Xie et al., 2023).

### RNA extraction and cDNA synthesis

Total RNA was extracted from leaves of Arabidopsis using the Easy Plant RNA Rapid Extraction Kit (Yiside Biotechnology, Hangzhou, China) according to the manufacturer’s instructions. First-strand cDNA was synthesized using the Hifair II 1st Strand cDNA Synthesis Kit (Yeasen Biotechnology, Shanghai, China).

### Gene cloning and vector construction

For the construction of AtAAS expression vector, cDNA synthesized from total RNA of Arabidopsis was used as the template, and the coding sequences were amplified in three overlapping fragments. The PCR fragments were then assembled and inserted into the NcoI-linearized pET-41b vector by homologous recombination. The construction was verified by Sanger sequencing. The primers used in this study were listed in **Supplementary Table S1**.

### Recombinant protein expression and purification

The constructed pET-41b derivatives were transformed into *E. coli* OverExpress C43(DE3) competent cells. A single colony was inoculated into 5 mL LB medium containing 50 μg/mL kanamycin and cultured overnight at 37°C with shaking (200 rpm). The overnight culture was then diluted 1:100 into 300 mL fresh LB medium with kanamycin and grown at 37°C until OD600 reached 0.6–0.8. Protein expression was induced by adding 100 μM IPTG, and the culture was further incubated at 23°C with shaking (180 rpm) for 12–14 h. Cells were harvested by centrifugation (4°C), resuspended in 60 mL PBS buffer, and lysed using a high-pressure homogenizer. The lysate was centrifuged at 9000 rpm for 20 min at 4°C, and the supernatant was collected. One milliliter of pre-equilibrated GST resin was added to the supernatant and incubated at 4°C for 1–2 h with gentle agitation. The mixture was then loaded onto an affinity column. After the flow-through had drained, the resin was washed three to five times with PBS buffer. Bound proteins were eluted five times with 1 mL of GST elution buffer (50 mM Tris-HCl, 10 mM reduced glutathione, pH 8.0). The eluted proteins were stored at –20°C. Protein purity was assessed by SDS-PAGE (10 μL sample mixed with 2.5 μL 5× loading buffer, heated at 98°C for 10 min, then run at 200 V for about 30 min). Gels were stained with FastBlue protein staining solution for 1–2 h and destained in water overnight before imaging.

### *In vitro* enzyme activity assays

The standard reaction mixture (100 μL) contained 50 mM Tris-HCl (pH 7.4), 0.5 mM substrate (tryptophan or 5-HTP), 0.1 mM PLP, and 50 μg of purified recombinant protein. The reaction was carried out at 30°C for 3 hours, then stopped by adding 200 μL methanol to precipitate proteins. Control reactions containing heat-denatured recombinant protein were performed in parallel under otherwise identical reaction conditions to exclude non-enzymatic product formation and background signals. After centrifugation at 10,000 rpm for 2 min, the supernatant was filtered through a 0.22 μm syringe filter and used for HPLC analysis. Kinetic parameters (Km and Vmax) for 5-HTP were determined by varying substrate concentrations from 0.05 to 30 mM. The data were fitted to the Michaelis-Menten equation using Prism 9 (GraphPad) to obtain the kinetic parameters. All assays were run in triplicate.

### Quantification of tryptamine and serotonin *in vitro* reactions

For the quantification of tryptamine, HPLC-FLD method was performed on a Shimadzu LC-20 system equipped with a fluorescence detector. Tryptamine was separated on a Welch Ultimate^®^ XB-C18 column (5 μm, 4.6 × 150 mm) at 35°C with an isocratic mobile phase of 0.1% formic acid/methanol (80:20, v/v) at 1 mL/min. Fluorescence detection was performed at 280/348 nm. Serotonin was analyzed on an InertSustain C18 column (5 μm, 4.6 × 250 mm) at 20°C using the same mobile phase composition (0.1% formic acid/methanol). Isocratic condition was 97:3 (v/v) for 0–15 min. The flow rate was 1 mL/min and the injection volume 5 μL in all cases. Detection for serotonin was performed at excitation/emission wavelengths of 280/348 nm.

### Extraction and LC-MS/MS analysis of 5-HTP, serotonin, N-acetylserotonin and melatonin in Arabidopsis

For each biological replicate, all tissues except roots from four 22-day-old Arabidopsis plants were pooled and ground for extraction. Approximately 0.14 g of the powder was transferred into a 2 mL centrifuge tube, and 700 μL of methanol-water (v/v = 4:1, containing 0.1% formic acid) was added. The mixtures were extracted overnight at 4°C with gentle rotation, then centrifuged at 12,000 rpm for 5 min. The supernatant was filtered through a 0.22 μm organic-phase syringe filter and used for LC-MS/MS analysis of 5-HTP, serotonin, N-acetylserotonin and melatonin. An ultra-high performance liquid chromatography coupled to tandem mass spectrometry (UHPLC-MS/MS) system (ExionLC™ AD UHPLC-QTRAP 6500+, AB SCIEX Corp., Boston, MA, USA) was used to quantitate compounds. Separation was performed on a Waters HSS T3 column (2.1×150 mm) which was maintained at 40°C. The mobile phase, consisting of 5 mM ammonium acetate in (0.1% formate) water (solvent A) and (0.1% formate) acetonitrile (solvent B), was delivered at a flow rate of 0.30 mL/min. The solvent gradient was set as follows: initial 5% B, 2 min; 5-40% B, 4 min; 40-70% B, 7 min; 70-100% B, 7.5 min; 100-5% B, 9.1 min; 5% B, 11 min.

The mass spectrometer was operated in positive multiple reaction monitoring (MRM) mode for all four analytes. The optimized MRM transitions were: for 5-HTP, m/z 221.1 → 204.2; for serotonin, m/z 177.1 → 160.0; for N-acetylserotonin (NAS), m/z 219.2 → 160.2; for melatonin, m/z 233.1 → 174.1. The following source parameters were used: IonSpray Voltage 4500 V (positive), Sheath Gas 35 psi, Ion Source Temperature 550°C, Auxiliary Gas 50 psi, Collision Gas 55 psi. Quantification was performed using external standard curves prepared with authentic standards, and data were normalized to fresh weight (ng/g FW).

### Natural variation in AtAAS

Natural variation in the AtAAS gene was analyzed using the POLYMORPH 1001 web tool (https://1001genomes.org) of the 1001 Genomes Project. Visualization of the variant positions was performed in R.

### Statistical analysis

All experiments were performed with at least three independent biological replicates unless otherwise indicated. Data are presented as mean ± SD. Biological replicate numbers are indicated in the figure legends. Statistical significance was assessed using Student’s t-test (GraphPad Prism 9.0). A P value < 0.05 was considered significant, and P < 0.01 was considered highly significant. Asterisks indicate significant differences compared to the control group (* for P < 0.05, ** for P < 0.01, and *** for P<0.001).

## Data Availability

The original contributions presented in the study are included in the article/**Supplementary Material**. Further inquiries can be directed to the corresponding authors.
