# Supplementary material for: Functional characterization of AtAAS as a 5-hydroxytryptophan decarboxylase linking serotonin formation to melatonin metabolism in Arabidopsis thaliana
Source: Front Plant Sci. 2026 Sep 15;17:1961230. doi: 10.3389/fpls.2026.1961230 (PMC13619621; doi:10.3389/fpls.2026.1961230)
Supplement: Supplementary file 1 [file DataSheet1.docx]

**Figure S1. In vitro tryptophan decarboxylase activity assay of recombinant AtAAS.**

25  μg or 50 μg of AtAAS protein was incubated with 1 mM tryptophan in 50 mM Tris‑HCl buffer (pH 7.4) containing 0.5 mM PLP at 30 °C for 3 hours. Heat‑inactivated protein reactions were used as controls. Fluorescence detection was performed with excitation at 280 nm and emission at 348 nm.

**Figure S2. LC‑MS/MS and HPLC confirmation of serotonin as the product of AtAAS‑catalyzed 5‑HTP decarboxylation.** Product ion spectra of serotonin standard (upper panel) and the reaction product (lower panel). The characteristic fragment ion at m/z 160.01 is clearly detected in both.

**Figure S3. Endogenous 5‑hydroxytryptophan (5‑HTP) levels in wild‑type (WT) and *aas* mutant plants under normal growth conditions.**
LC‑MS/MS analysis was performed on aerial tissues of 22‑day‑old seedlings. (A) Representative mass spectrometric chromatograms of 5‑HTP standard. (B) Quantification of 5‑HTP content in black as the control. (C) Quantification of 5‑HTP content (ng/g fresh weight) in WT plants. (D) Quantification of 5‑HTP content (ng/g fresh weight) in *aas* mutant plants.

**Table S1. Primers used for constructing expression vectors**

| Primers | Sequence (5’→3) |
| --- | --- |
| AtAAS-F1 | acgacgacaagagtcccATGGAAAATGGAAGCGGGA |
| AtAAS-R1 | AGAAGTGGTCCCAACATTAGCACATA |
| AtAAS-F2 | TATGTGCTAATGTTGGGACCACTTCT |
| AtAAS-R2 | tcggatccccgatatccatggTTACTTGTGAAGCAAGTAAGATGCTTC |

Lowercase letters indicate homologous arm sequences.
